# Divining Rods

**Published:** 1857-06

**Authors:** 


### DIVINING RODS.

WITH wonder and awe in the days of our childhood have we stood gazing at a man walking over the ground with measured pace and serious countenance, holding firmly in either hand a prong of a peach-tree twig, uniting in a forked end, which was uppermost. As he passed along in a certain direction the upper end would turn downwards, fairly twisting a prong off in the fingers of some stout unbeliever. We were told that where the twig turned downwards, there was the water. There our well was dug, and there water was found. There was the fact, and off and on we have thought of it ever since, in even doubt whether it was an imposture, a coincidence, or a reality. A gentleman writes to the *Scientific American* from his southern plantation as late as March of the present year, that his great trouble hitherto has been the scarcity of water for domestic purposes. He employed a diviner, who designated a spot within twenty feet of his old brackish, useless well, and has now an abundant supply of good water; closing by saying, "If this is a humbug, I wish most sincerely that I could be frequently humbugged in a similar way."

In a previous part of his concise communication, he says: "I have seen the divining rod point to a bunch of keys, or a purse hidden under leaves, and therefore think it very likely that it may also indicate beds of ores," and this brings us plump up to the subject of so-called "*spiritualism*" in connection with a note from a namesake of ours to whom reference was made in the March number as having invented an ugly word, if he has never done anything else—"VOLAUROLOGY;" what a mouthful to pronounce—one has to go away down his own throat to get at it. It just takes two Dutchmen to manage that one word. The idea is that *something goes out of man with power*, and that it is this power which tips tables and can make fat people turn round like tops whether they will or no—an involuntary waltz *solo!* Never having been inside of a ball-room while the "performances" were going on, we do not know at this writing whether it takes two to make a waltz, or if one is competent to the transaction; it is the turning round operation we are alluding to—perhaps it is the polka.

Mr. Hall writes: "With regard to me, you say, if I have anything *new*, print it. I believe the *agencies* may be productive of good not yet understood—that murders can be found out, crime detected, and that mental philosophy will have to undergo a change; that thoughts may be read, distances overcome, health restored, and ores, lead, tin, silver, and gold, may be discovered without instruments, many feet under ground. These metals emit a peculiar something, an '*aura*,' which is not intercepted by the soil; this meets an 'aura' from sensitive minds, and they feel the different effects. Silver produces different sensations from gold, and gold from other metals. Electricity is known to penetrate the solid earth to the distance of ten feet. Over the graves of the newly dead, sensitives can see and *do feel* the decaying decomposition, despite the superposed earth. Baron Von Reichenbach, who has demonstrated the power of the magnet, and made it visible so as to be produced on the daguerreotype plate, has shown clearly that every object in nature throws out an *aura* peculiar to itself, and when these *auræ* come in contact with the cultivated perceptions of the highly sensitive patient, they are seen and felt.

"Water can be felt fifty feet under ground, and by parity of reasoning, metals ought to be, as their '*aura*' is equally powerful. This is one of the new things I want to bring out of the study of the subject. How far I shall succeed, remains for the future to disclose."

If all the other statements be as wholly and as unmistakably true as the last sentence of our enthusiastic and modest correspondent, we will throw all medicines to the dogs, which, by the way, we would have been glad to have done long ago, though we are sure they would have had more sense than to have taken it. Hitherto, we have thrown it to our patients; for mind, this *aura*, when it can be "geared up" and the "reins" adjusted, is to "restore health." But there is another item we feel more interested in: it is the "golden" part, which will point as certainly to the place of deposit, as the peach-tree twig does to fountains of water. We have not the slightest doubt in the world, that this *aura* will indicate the direction where the gold is found in some localities. Can't exactly say that this is the point of difficulty. It is the getting at the gold when you have found out where it is, which has bothered us most. The *aura* in Gotham would always point to Wall street, but the more Herculean job is to get it out of Wall street when it once gets there. Millions go there every year, which practically, and to all intents and purposes, stay there.

We beg leave to suggest to our correspondent a more euphonious name as the representative of his thoughts on the subject. Call it, for example, the "AURAL THEORY." There is a silvery sound in that, while it is more philosophical. There may be volition about the "aura" of intelligent men, but there is none with inanimate objects. We know our term is the most comprehensive, sounds better, and is, critically, more accurate.

Odors go out from all things. Magnetic power goes out of some articles. The armature attracts the needle. Electrical influences go out of some objects, and enter others with great power. After all, there is some truth in all things; and as to the "*Aural Theory*," we will wait and see, not wait and shut our eyes, for that power goes out of bodies there can be no doubt—that out of beauty's eyes, for example: we were struck dumb and blind by it ourselves once upon a time.

